# miRNA Associated With Glucose Transporters in Oral Squamous Cell Carcinoma: A Systematic Review

**DOI:** 10.7759/cureus.46057

**Published:** 2023-09-27

**Authors:** Priyadharshini R, Monal Yuwanati, Saravanan Sekaran, Senthilmurugan M

**Affiliations:** 1 Oral Pathology and Microbiology, Saveetha Dental College and Hospitals, Saveetha Institute of Medical and Technical Sciences, Saveetha University, Chennai, IND; 2 Prosthodontics, Saveetha Dental College and Hospitals, Saveetha Institute of Medical and Technical Sciences, Saveetha University, Chennai, IND; 3 Oral and Maxillofacial Surgery, Saveetha Dental College and Hospitals, Saveetha Institute of Medical and Technical Sciences, Saveetha University, Chennai, IND

**Keywords:** expression, glut 3, squamous cell carcinoma, mirna, glut1

## Abstract

Oral squamous cell carcinoma (OSCC) is a malignancy of the oral cavity with poor prognosis. Dysregulation in glycolytic pathways involving glucose transporters (GLUT) has been implicated in poor prognosis. Furthermore, GLUT expression in cancer cells is regulated by several miRNAs. However, there is a lack of data about miRNA involved in the regulation of GLUT in OSCC. The objective is to evaluate the role of miRNA in the regulation of GLUT in OSCC. Data sources include PubMed (MEDLINE), Scopus, and Web of Science. Studies evaluating the miRNA involved or associated with the regulation of GLUT in OSCC were included in the systematic review. Data pertaining to GLUT and associated miRNA expression were extracted from studies. Qualitative assessment was carried out for GLUT and miRNA. The Newcastle-Ottawa Scale was used for quality assessment. Ten study articles were included after analyzing 4675 papers. These studies evaluated the GLUT and miRNA expression between healthy and OSCC samples. There are variable expression patterns of GLUT in OSCC. Furthermore, it was dependent on miRNA. The GLUT1 and GLUT-3 were detected more frequently in OSCC, while no study reveals the expression of GLUT2, GLUT4, GLUT7, GLUT8, GLUT13, SGLT1, and SGLT2 with miRNA regulation. However, there was insufficient evidence on specific miRNA linked to GLUT1 or GLUT3 expression. There is evidence of the role of miRNA in the regulation of GLUT especially GLUT1 and GLUT3 in OSCC; however, a specific relation to miRNA was understudied. In the future, studies exploring a clearer understanding of the association between miRNA and the GLUT metabolic pathway in relation to OSCC are warranted. Furthermore, association of miRNA and GLUT with progression of disease, disease resistance, and prognosis is assessed for better treatment outcomes.

## Introduction and background

Oral squamous cell carcinoma (OSCC) is a major global, chronic, non-communicable disease responsible for millions of deaths every year with the worst morbidity and mortality causing psychological and financial debility among cancer patients. Surgery, chemotherapy, and radiation are common modalities for treating OSCC. Furthermore, immunomodulatory drugs are developed to improve survival and mortality [1]. However, irrespective of it, survival and mortality have not shown much improvement. Uncontrolled cell proliferation is one of the hallmarks of cancer. Malignant cells undergo rapid proliferation which needs utilization of glucose for the synthesis of ATP. Cellular uptake of glucose is regulated by cell surface proteins such as glucose transporters (GLUT) and sodium glucose-linked transporters (SGLT). Previous studies have found increased expression of GLUT and SGLT in malignant cells [2].

Hypoxemic areas regulate hypoxia-induced factor (HIF1α) with an increase in GLUT1 expression and uptake by shifting glucose metabolism to glycolysis. Furthermore, GLUT1 expression in tumors leads to poor prognosis with increased production of lactate. Higher affinity of GLUT1 provides energy in hypoxic environments that influence the rate of growth, aerobic environment, and malignant formation in the tumor microenvironment which leads to a shorter overall survival rate in OSCC [3]. miRNA are small endogenous non-coding molecules that regulate the target binding of mRNAs. miRNAs play a major role in differentiation, proliferation, apoptosis, recurrence, epithelial-mesenchymal interaction, and metastasis [4]. Inactivation of miRNAs by CpG island hypermethylation was considered an epigenetic modification in carcinoma. miRNA silencing suppresses the mTOR-AKT signaling pathway during carcinogenesis with resultant alteration in metabolism followed by proliferation of tumor cells [5], and miRNAs were repressed in case of disease showing impaired prognosis [6].

miRNA expression influences the GLUT1 expression with a subsequent lactate production and increased glucose secretion and uptake. Altered glycolytic regulation by miRNAs plays a key role in proliferation of cancer cells through paracrine functions among the neighboring tumor microenvironment facilitating significant apoptosis of immune cells and angiogenesis which contribute to the hallmarks of cancer [7]. Warburg in 1900 described the glycolysis for ATP generation in an oxidative environment by cancer cells for its growth. The process of this glycolysis under aerobic conditions is called the Warburg effect [8]. Hypoxia-driven GLUT1 expression reveals an anti-stromal pattern in regions that were devoid of squamous keratinization [9].

Dysregulation in glycolytic pathways is the main hallmark of cancer. GLUT1 manifestation in anaerobic glycolysis and hypoxia of tissues promote tumor growth [10] and its survival; hence, GLUT expression alteration is reported in premalignancy and malignancy with altered GLUT1 responses in relation to the clinical staging and histopathological grading of OSCC as a prediction revealed based on its aggressiveness and prognosis [11,12]. As sufficient data explaining the role of various miRNA were inadequate, the study aimed to analyze the role of various miRNA associated with GLUT in OSCC.

## Review

Review methods

The present systematic review was reported according to Preferred Reporting Items for Systematic Reviews and Meta-Analyses (PRISMA) 2020 guidelines [13].

Eligibility criteria

The papers were screened for eligibility based on the following inclusion criteria and exclusion criteria.

Inclusion criteria

Studies evaluating GLUT expression in OSCC and normal tissue, studies evaluating the miRNA expression in OSCC and normal tissue, studies that have provided data on miRNA and GLUT expression in OSCC and normal tissue, and only studies published in the English language were included.

Exclusion criteria

Studies that have evaluated miRNA and GLUT expression on precancerous and other malignant lesions, case reports, editorials, letters, expert reviews, and articles were excluded.

Search strategy and information sources

A comprehensive search was performed in PubMed, Scopus, and Web of Science repository datasets to retrieve the relevant articles (from inception to March 31, 2023). The curated search strategy was used to retrieve relevant studies data from databases with the below-mentioned keywords: ((“OSCC” OR “Oral cancer” OR “Oral carcinoma” OR “Head & Neck neoplasm” OR “Mouth cancer” OR “SCC”) AND (“miRNA” OR “mRNA” OR “microRNA”) AND (“GLUT” OR ”Glucose transporter” OR “SGLT” OR “GLUT 1”)). 

Screening and selection process

The papers retrieved during search were screened for potential eligibility as per criteria. These papers were initially screened after reading the title, abstract, and keywords followed by full-text reading of filtered papers. Screening of the collected data was done by two blinded independent reviewers (RP, MY). In case of disagreement on the selection of articles for inclusion between two reviewers, a third reviewer was consulted, and the final decision was taken by the third reviewer.

Data items

Two reviewers extracted the data in duplicate using the predefined format in a Microsoft Excel spreadsheet (Microsoft Office 2011, 32-Bit Edition, Microsoft Corporation) with an agreement of 0.85. The data collected include the author, year, country, type of sample, number of samples, type of miRNA and GLUT, miRNA and GLUT expression, and effect of miRNA expression on the tumor tissue/cell line (Table 1). In case of disagreement on data such as the number of samples, expression pattern, expression values, etc., between two reviewers, a third reviewer was consulted to resolve it. 

**Table 1:** Risk of Bias Assessment (Newcastle-Ottawa Scale) A maximum of one star can be assigned to each numbered item within the selection and exposure categories for case-control studies, or the selection and outcome categories for cohort studies. In both types of studies, a maximum of two stars can be awarded for comparability, resulting in a total maximum score of nine points.

Author	Year	Selection	Compatibility	Outcome	Total Score
Chen et al., [16]	2019	Four star	One star	Two star	Good quality
Wang et al., [17]	2018	Four star	One star	Two star	Good quality
Guo et al., [18]	2020	Four star	One star	Two star	Good quality
Xu et al., [19]	2015	Four star	One star	Two star	Good quality
Chen et al., [20]	2019	Four star	One star	Two star	Good quality
Xu et al., [21]	2018	Four star	One star	Two star	Good quality
Li et al., [22]	2020	Four star	One star	Two star	Good quality
Yan et al., [23]	2018	Four star	One star	Two star	Good quality
Yang et al., [24]	2021	Four star	One star	Two star	Good quality
Wang et al., [25]	2020	Four star	One star	Two star	Good quality

Risk of bias and quality assessment

Risk of bias was assessed using the NOS for non-randomized studies [14]. Studies were assessed in selection, comparability, and outcome/exposure domains. Each question was given either one (selection and exposure/outcome domain) or two stars (comparability domain). A study score >6 was considered a good-quality study. Assessment was carried out by two reviewers to avoid bias and agreement was 0.78. Disagreements were discussed and resolved through consensus. 

Summary synthesis

The studies were summarized using descriptive statistics. Due to heterogeneity of data, meta-analysis was not carried out. The summary synthesis was reported according to Synthesis without meta-analysis (SWiM) in systematic reviews [15]. The studies were grouped based on miRNA, GLUT, and in vivo or in vitro, animal, and human miRNA involved in GLUT regulation in OSCC.

Results

Search Results

**Figure 1:**
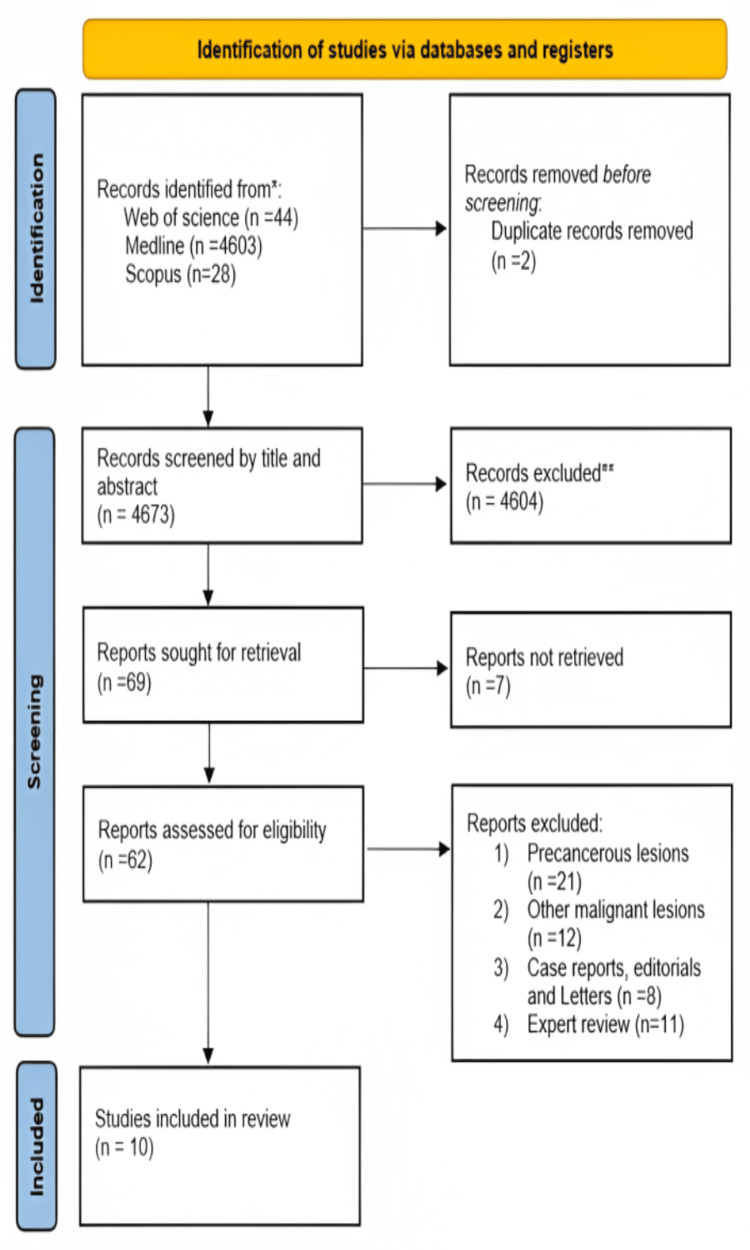
PRISMA chart PRISMA: Preferred Reporting Items for Systematic Reviews and Meta-Analyses

Relevant evidence from search data was retrieved from Web of Science, PubMed (MEDLINE), and Scopus. A total of 4675 papers were obtained during database search. Two duplicates were removed from these papers. After the title and abstract screen, only 69 papers (4604 papers excluded) were selected from full-text screening. Finally, 10 papers were found eligible for inclusion in the systematic review after the exclusion of 42 papers (two duplicates, 22 precancerous lesions, 12 other malignant lesions, eight case reports, editorials, and 10 expert reviews) (Figure 1).

Study Characteristics

All included studies were ex vivo and from China. These studies were conducted between 2015 and 2021 based on ex vivo cell line finding and samples of human tumor specimens and four animal-based study findings. The results were further stratified based on 10 studies with miRNA expression, type of glucose transporters, and its characterization.

Glucose transporters and miRNA expression in tissue and cells

There was a total of 10 studies (947 samples) showing correlation of GLUT with miRNA (Table 2) out of which five studies showed upregulated expression of miRNA directly proportional to the increase in glycolysis resulting in proliferation of tumor tissues via GLUT 1 regulation [16-20]. One study showed miRNA downregulation inversely proportional to the rise in glycolysis related to GLUT1 resulting in tumor growth promotion [21]. Three studies revealed that downregulation of miRNA leads to a resultant decrease in the action of glucose transporters via GLUT1 which inhibited the growth of cancer [22-24], while one GLUT 3 study contradicted the GLUT 1 study stating that upregulated miRNA inhibited the growth due to downregulation of GLUT expression [25].

**Table 2:** Characteristics of the studies included in the systematic review. A total of 10 studies were included after screening the literature about the miRNA expression pattern, type of GLUT transporter in tumor tissue, cell line, and its findings GLUT: Glucose transporter

Author	Year	Country	Type of Sample	Sample	Type of glucose transporter	Type of miRNA	Mean miRNA (Fold change)	Mean GLUT (Fold change)	miRNA Expression	GLUT Expression	Effect of miRNA expression on Tumor tissue/Cell line
Chen et al.,[16]	2019	China	Tumor tissue Cell line	20 OSCC CAL‐27, Tca8113	GLUT1,	miRNA-378a	Normal-2.2 Tumour-4.2	Normal-1.7 Tumour-1.2	Upregulated	Downregulated	Promotes tumor growth
Wang et al., [17]	2018	China	Tumor tissue Cell line	49OSCC HSC-3, HSC-4, HSC-6, CAL-27, and HEK 293T	GLUT 1	miRNA-378-3p	Normal-1 Tumour-1.4	Normal-1 Tumour-0.2	Upregulated	Downregulated	Promotes tumor growth
Guo et al., [18]	2020	China	Tumour tissue Cell line	60 OSCC CAL-27, SCC-4, SCC-9 and SCC-25	GLUT 1	miRNA-182-5p	Normal-0.7 Tumour-0.5	Normal-0.5 Tumour-0.4	Downregulated	Downregulated	Promotes tumor growth
Xu et al., [19]	2015	China	Tumour tissue Cell line	3 OSCC SCC15, SAS carcinoma cells and HEK293 T cells	GLUT 1	miRNA-340	Normal-1 Tumour-0.5	Normal-1 Tumour-0.8	Downregulated	Downregulated	Inhibits tumor growth
Chen et al., [20]	2019	China	Tumour tissue Cell line	52 OSCC SCC090 and SCC25	GLUT 1	miRNA‑10a	Normal-1.9 Tumour-3.1	Normal-1.8 Tumour-3.2	Upregulated	Upregulated	Promotes tumor growth
Xu et al., [21]	2018	China	Tumor tissue Cell line	68OSCC SCC09 cell, SCC15, SCC25, PCS-200-014	GLUT 1	miRNA-218			Downregulated	Upregulated	Promotes tumor growth
Li et al., [22]	2020	China	Tumor tissue Cell line	51 OSCC SCC-25 and CAL-27	GLUT 1	miRNA-150-5p			Upregulated	Downregulated	Promotes tumor growth
Yang et al., [23]	2018	China	Tumor tissue Cell line	78 OSCC SCC090 and SCC 25	GLUT 1	miRNA 200c			Upregulated	Downregulated	Promotes tumor growth
Yang et al., [24]	2021	China	Tumor tissue Cell line	528 OSCC Cal-27, UM SCC-1, HSC-2 and FaDu	GLUT1	miRNA-675-5p			Downregulated	Upregulated	Promotes tumor growth
Wang et al., [25]	2020	China	Tumor tissue Cell line	10 OSCC Detroit562 and Eca-109	GLUT 3	miRNA-23a-3p			Upregulated	Downregulated	Inhibits tumor growth

Discussion

Our review found that miRNA plays a role with GLUT in OSCC and elucidates the association of miRNA with GLUT in OSCC. GLUT and miRNA overexpression relative to the normal oral keratinocyte cell line were observed in seven cell line studies [16-19,21,24]. However, two studies showed down regulated expression of miRNA and GLUT [22,23]. Out of detailed 10 studies on GLUT level expression with the specific miRNA, only six studies have demonstrated the upregulated expression of GLUT1 on patient tumor study samples (438/778) with the majority of studies analyzing that altered miRNA-associated expression through increased GLUT1 uptake is responsible for tumor progression through cell proliferation, invasion, and metastasis when correlated with the advanced tumor stage but it did not show any correlation with overall survival after the relapse-free survival of the disease [16-20,25] and four studies have demonstrated the down expression of GLUT1 in patient’s tumor samples (135/159) [21-24]. Of these studies, 215 patients showed positive correlation with GLUT1-associated proliferation and differentiation of tumors [16,19-21,23,24].

Wang et al. in their 2020 study observed an upregulation of GLUT3 expression in the cell line, which was associated with the suppression of glucose uptake in tumor tissue samples (6/10) compared to healthy tissues. Furthermore, poor survival was linked to increased GLUT1 uptake due to SIX1 upregulation, establishing it as a prognostic factor for oral carcinoma. Interestingly, there is only one study that explains the role of miRNA in cancer proliferation and metastasis through GLUT3 uptake, and it appears to support disease-free survival, reduce relapse time for oral carcinoma, and improve overall survival among patients [25]. In their 2019 study, Chen and colleagues discovered that Circ_100290 mediated the silencing of miRNA378a, leading to the upregulation of GLUT1, which, in turn, facilitated increased glycolysis and cell proliferation [16]. In 2018, Wang demonstrated that the overexpression of miRNA 378a-3p induces proliferative signaling in OSCC cells by modifying epithelial-mesenchymal transition, matrix metalloproteinase expression, and cellular communication network factors, ultimately resulting in enhanced cell proliferation [17]. Elevated glycolysis led to enhanced cell proliferation in OSCC, and this effect was positively associated with increased GLUT1 expression via miRNA-10a, as compared to adjacent control tissues. A previous investigation by Guo in 2020 had already reported that elevated miRNA-10a expression was linked to the progression and prognosis of lung cancer [18].

In the study conducted by Xu et al. in 2020, they observed that miRNA-150-5p exhibited reduced expression, which was inversely related to Pvt-1 expression. This reduction in miRNA-150-5p, along with an increase in GLUT1 expression, led to the proliferation, migration, and invasion of tumor cells. Additionally, when Pvt-1 was knocked down in mice, it was associated with a poor prognosis for OSCC [19]. In 2019, Chen and colleagues found that miRNA-200c inhibits the proliferation of tumor tissues by targeting Akt, which subsequently leads to the downregulation of GLUT1 downstream [20]. In their 2018 study, Xu et al. demonstrated that decreased expression of miRNA-218 correspondingly elevates the expression of GLUT1 in tumor tissues during the advanced stages of tumor development, contributing to the progression of the tumor [21]. Conversely, in the study conducted by Li et al. in 2020, overexpression of Circ_0000140 intensified the downregulation of miRNA-182-5p, leading to a subsequent reduction in glucose metabolism through lactate dehydrogenase (LDHA) and resulting in diminished growth of tumor cells [22]. In their 2018 study, Yan and colleagues found that a shift in metabolic mediators associated with miRNA-340 contributes to a resulting decrease in GLUT1-mediated glycolysis and a reduction in the proliferation of tumor cells. This reduction is achieved by the inhibition of GLUT1 expression through sequence binding in the 3’-UTR.

In 2021, Yang and colleagues discovered that miRNA-675-5p amplifies the glycolytic pathway, leading to the proliferation of cancer-associated fibroblasts and consequently inhibiting tumor growth in malignancies such as oral cancer. Additionally, miRNA-23a-3p was found to target the SIX1/GLUT3 axis, suppressing the maturation of OSCC and reducing GLUT3 uptake [25]. A similar study on various cancer types explores how miRNA and the regulation of glucose transporters contribute to promoting cancer growth and metastasis [26-28]. In their 2010 study, Ayala et al. highlighted that the precise pathway linking GLUT 1&3 with specific miRNA and their relationship with tumor grading and staging, ultimately resulting in metastasis, remained uncertain. This suggests that tumor progression is not solely dependent on glucose transporters but can also be influenced by risk factors such as smoking, alcohol consumption, viral infections, and poor oral hygiene [29].

The study constraints underscore the necessity for research that inhibits specific miRNA molecules associated with GLUT and their impact on tumor advancement. This review is marked by a scarcity of literature references, a lack of comprehensive information regarding squamous cell carcinoma grades and their correlation with GLUT and miRNA, a limited number of cancer samples in the studies for comparative analysis, and an absence of investigation into the expression of additional glucose transporters such as GLUT2, GLUT4, GLUT7, GLUT8, GLUT13, SGLT1, and SGLT2 in conjunction with miRNA regulation in OSCC, all of which are identified as shortcomings.

## Conclusions

There is evidence of the role of miRNA in regulation of GLUT especially GLUT1 and GLUT3 in OSCC; however, a specific relation to miRNA was understudied. Future research has to be done for clearer understanding of the GLUT metabolic pathway in relation to OSCC. Further association of GLUT with progression of disease, disease resistance, and prognosis needs to be delineated.
